# Brain grey matter volume alterations associated with antidepressant response in major depressive disorder

**DOI:** 10.1038/s41598-017-10676-5

**Published:** 2017-09-05

**Authors:** Jia Liu, Xin Xu, Qiang Luo, Ya Luo, Ying Chen, Su Lui, Min Wu, Hongyan Zhu, Graham J. Kemp, Qiyong Gong

**Affiliations:** 1 Huaxi MR Research Center (HMRRC), Department of Radiology, West China Hospital of Sichuan University, Chengdu, China; 2 Department of Psychiatry, West China Hospital of Sichuan University, Chengdu, China; 3 Laboratory of Stem Cell Biology, State Key Laboratory of Biotherapy, West China Hospital of Sichuan University, Chengdu, China; 4 Institute of Ageing and Chronic Disease, Faculty of Health and Life Sciences, University of Liverpool, Liverpool, United Kingdom; 5 Liverpool Magnetic Resonance Imaging Centre (LiMRIC), University of Liverpool, Liverpool, United Kingdom

## Abstract

Not all patients with major depressive disorder respond to adequate pharmacological therapy. Psychoradiological studies have reported that antidepressant responders and nonresponders show different alterations in brain grey matter, but the findings are inconsistent. The present study reports a meta-analysis of voxel-based morphometric studies of patients with major depressive disorder, both antidepressant responders and nonresponders, using the anisotropic effect size version of Seed-based D Mapping to identify brain regions correlated to clinical response. A systematic search was conducted up to June 2016 to identify studies focussing on antidepressant response. In responders across 9 datasets grey matter volume (GMV) was significantly higher in the left inferior frontal gyrus and insula, while GMV was significantly lower in the bilateral anterior cingulate cortex (ACC) and the right superior frontal gyrus (SFG). In nonresponders across 5 datasets GMV was significantly lower in the bilateral ACC, median cingulate cortex (MCC) and right SFG. Conjunction analysis confirmed significant differences in the bilateral ACC and right SFG, where GMV was significantly lower in nonresponders but higher in responders. The current study adds to psychoradiology, an evolving subspecialty of radiology mainly for psychiatry and clinical psychology.

## Introduction

Major depressive disorder (MDD) accounts for a large burden of disease, and is a leading cause of years lived with disability^1^. Antidepressant medication is a first-line treatment for severe MDD^2^, and has been shown to ameliorate functional impairment, with changes in neural activation and brain structure^3, 4^. However, ~30% of patients do not respond to adequate pharmacological therapy, and the pathophysiological mechanisms linking depression, structural change and treatment response remain unclear^5–7^.

Although antidepressant responders and nonresponders show grey matter volume (GMV) alterations by structural magnetic resonance imaging (MRI)^8–10^, reports relating to antidepressant effects are inconsistent. For example, higher GMV in the right superior temporal gyrus was reported in one study of responders^11^, while lower GMV in the right superior frontal gyrus of nonresponders has been observed in some studies^8, 9, 12^, but not others^13^. Variations in sample sizes, imaging protocols, and the demographic and clinical characteristics of the patients may underlie much of this inconsistency. Meta-analysis therefore offers a valuable way to define consistent GMV abnormalities in MDD responders and nonresponders, to throw light on the pathophysiological mechanisms underlying antidepressant effects.

The automated analysis method of voxel-based morphometry (VBM) provides a powerful tool to compare group differences in GMV at whole-brain level^14^. To identify consistent regional GMV abnormalities in relation to antidepressant effect, both positive and negative results of VBM studies can be combined in the same map by using a particular voxel-based meta-analytic approach, the Anisotropic Effect Size version of Seed-based D Mapping (http://www.sdmproject.com, AES-SDM). AES-SDM supports effect size comparison and conjunction analysis^15^, and has been used to compare MDD with bipolar disorder^16, 17^ and in other neurologic disorders such as migraine^18^ and dementia^19^.

Using AES-SDM, this systematic meta-analysis aimed to (1) investigate morphometric changes in MDD responders and nonresponders compared with healthy controls, and (2) compare GMV differences that may define specific and shared morphological alterations in responders and nonresponders.

## Results

### Included studies and their characteristics

We found 2512 studies, of which 10 studies^4, 8, 9, 11–13, 20–23^ ultimately met the inclusion criteria. No additional study was identified from their references. Figure S1 shows a flow diagram of study selection. This left a total of 10 articles for our meta-analysis, with responders across 9 datasets (199 patients vs. 308 controls) and nonresponders across 5 datasets (120 patients vs. 132 controls). Table 1 summarises the clinical characteristics of these groups in the various studies.

**Table 1:** Characteristics of patient and control groups in studies included in the meta-analysis.

Studies	Number (female)		Mean age, y		Severity (scale type)		Illness duration (months)	Scan time	Antidepressants	MRI	Quality score
	MDD	HC	MDD	HC	Pre-tx	Pre-tx					
**Responders**											
Fang *et al*.^11^	20 (8)	18(8)	59.2 (3.7)	59.1 (7.5)	26.6(1.9) (HDRS)	6.4(0.98) (HDRS)	43.2(13.2)	Post-tx	Unclear	1.5 T	12
Jung *et al*.^12^	24 (17)	29(21)	43.0 (10.1)	43.6 (13.4)	20.9(4.1) (HDRS)	7.3(2.6) (HDRS)	33.3(43.1)	Pre-tx	SSRI/SNRI/others*	3 T	12.5
Klauser *et al*.^5^	27 (18)	33(21)	35.0 (9.72)	34.7 (9.93)	NA	13.04(11.73) (BDI)	108.48(78.36)	Post-tx	SSRI/SNRI/others	1.5 T	12
Kong *et al*.^20^	24 (14)	28(14)	36.1 (5.73)	32.1 (9.27)	NA	3.42(2.55) (HDRS)	4.12(0.89)	Post-tx	Fluoxetine	1.5 T	12.5
Li *et al*.^13^	19 (13)	25(19)	42.6 (13.0)	40.6 (12.7)	21.0(4.0) (HDRS)	3.4(2.0) (HDRS)	108(78.36)	Pre-tx	SSRI/SNRI/others	1.5 T	12
Liu *et al*.^22^	19 (19)	19(19)	37.6 (12.7)	36.8 (11.2)	NA	4.63 (HDRS)	88.44(66.4)	Post-tx	SSRI/SNRI/others	3 T	11.5
Ma. *et al*.^21^	17 (7)	17(7)	26.7 (7.73)	24.2 (4.41)	25.58(6.32) (HDRS)	NA	2.59(1.33)	Pre-tx	SSRI/SNRI/TCA	1.5 T	11
Salvadore *et al*.^23^	27 (21)	107(60)	40.2 (12.2)	36.2 (0.3)	NA	1.6(2.3) (MADRS)	181.2(164.4)	Post-tx	Unclear	3 T	12
Serra-Blasco *et al*.^9^	22 (20)	32(23)	48.0 (8.7)	46.0 (8.3)	NA	4(5.2) (HDRS)	214.3(129)	Post-tx	SSRI/SNRI/others	3 T	12
**Nonresponders**											
Jung *et al*.^12^	26 (19)	29(21)	40.8 (12.7)	43.6 (13.4)	18.8(4.7) (HDRS)	14.9(4.6) (HDRS)	36.4(33.2)	Pre-tx	SSRI/SNRI/others	3 T	12.5
Li *et al*.^13^	25 (20)	25(19)	46.5 (10.4)	40.6 (12.7)	21.9(3.5) (HDRS)	16.3(6.0) (HDRS)	112.8(102)	Pre-tx	SSRI/SNRI/ others	1.5 T	12
Ma. *et al*.^21^	18 (7)	17(7)	27.4 (7.74)	24.2 (4.41)	23.89(3.69) (HDRS)	NA	35.5(49.89)	Pre-tx	SSRI/SNRI/TCA	1.5 T	11
Machino *et al*.^8^	29 (13)	29(13)	39.6 (8.29)	38.7 (8.36)	NA	13.9(4.33) (HDRS)	52.55	Post-tx	SSRI/SNRI/ others	1.5 T	11
Serra-Blasco *et al*.^9^	22 (18)	32(23)	49.0 (8)	46.0 (8.3)	NA	21(4.6) (HDRS)	271	Post-tx	SSRI/SNRI/others	3 T	12

BDI = Beck Depression Inventory; HC = healthy controls; HDRS = 17-item Hamilton Depression Rating Scale; m = months; MADRS = Montgomery and Åsberg Depression Rating Scale; MAOI = monoamine oxidase inhibitors; NaSSA = noradrenergic/specific serotonergic agents; NDRI = norepinephrine dopamine reuptake inhibitor; Pre-tx = pre-treatment; Post-tx = post-treatment; SSRI = specific serotonin reuptake inhibitors; SNRI = serotonin norepinephrine reuptake inhibitors; TCA = tricyclic antidepressants; y = years; *others including TCA, NDRI, NaSSA and MAOI; All data are given as the mean (SD) if not otherwise specified.

### Antidepressant responders and nonresponders vs. healthy controls

**Figure 1:**
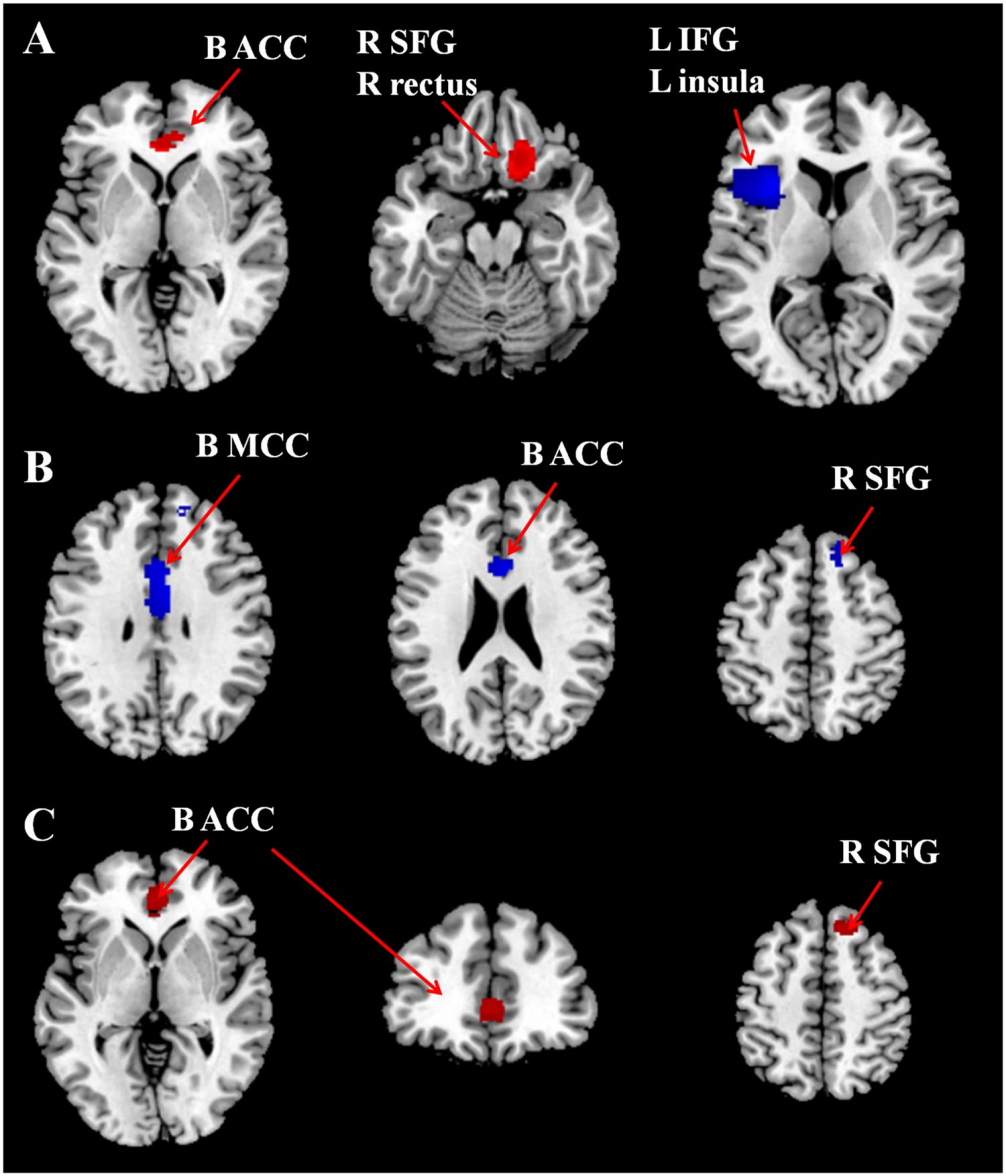
Regional differences in grey matter volume in antidepressant responders and nonresponders vs. healthy controls and antidepressant nonresponders vs. responders by meta-analysis. The figure shows areas of lower (blue) and higher (red) grey matter volumes in (**A**) responders and (**B**) nonresponders compared with healthy controls, and nonresponders compared with responders (**C**). Abbreviation: ACC = anterior cingulate cortex; B = bilateral; IFG = inferior frontal gyrus; L = left; MCC = median cingulate cortex; R = right; SFG = superior frontal gyrus.

Table 2 and Fig. 1 show the results of meta-analysis of both patient groups against healthy controls.

**Table 2:** Regional differences in grey matter volume in antidepressant responders and nonresponders compared with healthy controls

Brain Regions	Maximum			Clusters	
	MNI coordinates, x,y,z	SDM value	p-value	No. of voxels	Breakdown (no. of voxels)
**Responders > HC**					
Right frontal orbito-polar tract	18, 30, −18	2.321	0.000055730	411	R superior frontal gyrus, orbital part, BA 11 (157)
					R gyrus rectus, BA 11 (112)
					R frontal orbito-polar tract (93)
					R inferior frontal gyrus, orbital part, BA 11 (49)
Corpus callosum	6,34,2	2.221	0.000240505	333	R anterior cingulate cortex, BA 10,11,24,25,32 (152)
					L anterior cingulate cortex, BA 10,11,24,25,32 (131)
					Corpus callosum (50)
**Responders < HC**					
L insula, BA 48	−32,16,8	−1.658	0.000001013	909	L insula, BA 45,47 (451)
					L inferior frontal gyrus, BA 44,45,47,48 (262)
					L superior longitudinal fasciculus III (100)
					Left temporal pole, superior temporal gyrus, BA 48 (50)
					Left frontal aslant tract (23)
					Left lenticular nucleus, putamen, BA 48 (23)
Left inferior frontal gyrus, BA 48	−36,14,26	−1.019	0.001406848	42	L inferior frontal gyrus, BA 44,48 (42)
					L frontal inferior longitudinal fasciculus (15)
**Subgroup analysis**					
**Responders post-tx > HC**					
Right superior frontal gyrus, BA 11	20,30,−16	2.287	0.000110447	345	R superior frontal gyrus, orbital part, BA 11,25 (149)
					R gyrus rectus, BA 11 (82)
					R frontal orbito-polar tract (64)
					R inferior frontal gyrus, orbital part, BA 11 (50)
Corpus callosum	−4,34,2	2.280	0.000156879	265	R anterior cingulate cortex, BA 10,11,24,25,32 (106)
					L anterior cingulate cortex, BA 10,11,24,25,32 (107)
					Corpus callosum (52)
**Responders post-tx < HC**					
Left superior longitudinal fasciculus III	−34,20,12	−1.566	0.000009298		L insula, BA 45,47(144)
					L inferior frontal gyrus, BA 44,45,47,48 (140)
					L superior longitudinal fasciculus III (75)
Left frontal inferior longitudinal fasciculus	−36,20,22	−1.138	0.000341654		L inferior frontal gyrus, BA 44,48 (55)
					Left frontal inferior longitudinal fasciculus (23)
**Nonresponders > HC**	—	—	—	—	—
**Nonresponders < HC**					
Corpus callosum	6,6,28	−2.246	0.000132143	572	R median cingulate cortex, BA 24,23 (146)
					L median cingulate cortex, BA 24,23 (128)
					L anterior cingulate cortex, BA 24 (97)
					Corpus callosum (87)
					Left median network, cingulum (65)
					R anterior cingulate cortex, BA 24 (49)
R superior frontal gyrus, BA 9	16,38,46	−1.899	0.000587285	152	R superior frontal gyrus, BA 8,9 (122)
					Corpus callosum (30)
Right superior frontal gyrus, medial, BA 10	2,48,2	−1.742	0.001101315	87	L anterior cingulate cortex, BA 10,32 (52)
					R superior frontal gyrus, medial, BA 10 (23)
					R anterior cingulate cortex, BA 10,32 (12)
**Responders > Nonresponders**					
R anterior cingulate/paracingulate gyri	2,42,0	2.857	<0.001	283	
L anterior cingulate/paracingulate gyri	0,30,20	1.165	<0.001	43	
R superior frontal gyrus	4,58,4	2.024	<0.001	744	
**Responders < Nonresponders**					
—	—	—	—	—	

BA = Brodmann area; HC = healthy controls; L = left; M = middle; MNI = Montreal Neurological Institute; Post-tx = post-treatment; R = right; SDM = signed differential mapping.

Group comparison of responders against healthy controls revealed higher GMV in the bilateral anterior cingulate cortex (ACC), the right superior frontal gyrus (SFG) and gyrus rectus, and lower GMV mainly in the left inferior frontal gyrus (IFG) and insula. Results from subgroup analysis were consistent with these results.

Group comparison of nonresponders against healthy controls revealed lower GMV in the bilateral median cingulate cortex (MCC), ACC, and right SFG. There was no higher GMV in the nonresponders.

### Antidepressant nonresponders vs. responders

Conjunction analysis found significant differences in the bilateral ACC and right SFG, where GMV was lower in nonresponders but higher in responders.

### Jack-knife sensitivity analysis

Tables 3 and 4 show the results of whole-brain jack-knife sensitivity analysis. In responders, higher GMV in the right SFG and lower GMV in the left IFG and insula were highly replicable, being preserved throughout all 9 combinations of the datasets; higher GMV in the bilateral ACC and right gyrus rectus were significant in all but 1 combination. In nonresponders, lower GMV in bilateral MCC, ACC and right SFG were significant in all but 1 combination of the data sets.

**Table 3:** Sensitivity analyses of voxel-based morphometric studies of grey matter in antidepressant responders compared with healthy controls.

Studies	Higher grey matter					Lower grey matter	
	R SFG	R GR	R IFG	R ACC	L ACC	L IFG	L insula
Fang, J. *et al*.^10^	Y	Y	Y	Y	Y	Y	Y
Jung, J. *et al*.^11^	Y	Y	Y	Y	Y	Y	Y
Klauser P. *et al*.^4^	Y	Y	Y	Y	Y	Y	Y
Kong, L. *et al*.^19^	Y	Y	Y	Y	Y	Y	Y
Li, C.T. *et al*.^12^	Y	Y	Y	Y	Y	Y	Y
Liu, *et al*.^21^	Y	Y	Y	Y	Y	Y	Y
Ma C. *et al*.^20^	Y	Y	Y	Y	Y	Y	Y
Salvadore, G. *et al*.^22^	Y	N	N	N	N	Y	Y
Serra-Blasco, M. *et al*.^9^	Y	Y	Y	Y	Y	Y	Y

ACC = anterior cingulate cortex; GR = gyrus rectus; IFG = inferior frontal gyrus; L = left; r = right; SFG = superior frontal gyrus.

**Table 4:** Sensitivity analyses of voxel-based morphometric studies of grey matter in antidepressant nonresponders compared with healthy controls.

Studies	Lower grey matter				
	L MCC	L ACC	R MCC	R ACC	R SFG
Jung, J. *et al*.^11^	Y	Y	Y	Y	Y
Li, C. *et al*.^12^	Y	Y	Y	Y	Y
Ma C *et al*.^20^	Y	Y	Y	Y	Y
Machino, A. *et al*.^8^	N	N	N	N	N
Serra-Blasco, M. *et al*.^9^	Y	Y	Y	Y	Y

ACC = anterior cingulate cortex; HG = Heschl gyrus; IFG = inferior frontal gyrus; L = left; LG = lingual gyrus; MCC = median cingulate cortex; SFG = superior frontal gyrus.

### Meta-regression analysis and publication bias analysis

**Figure 2:**
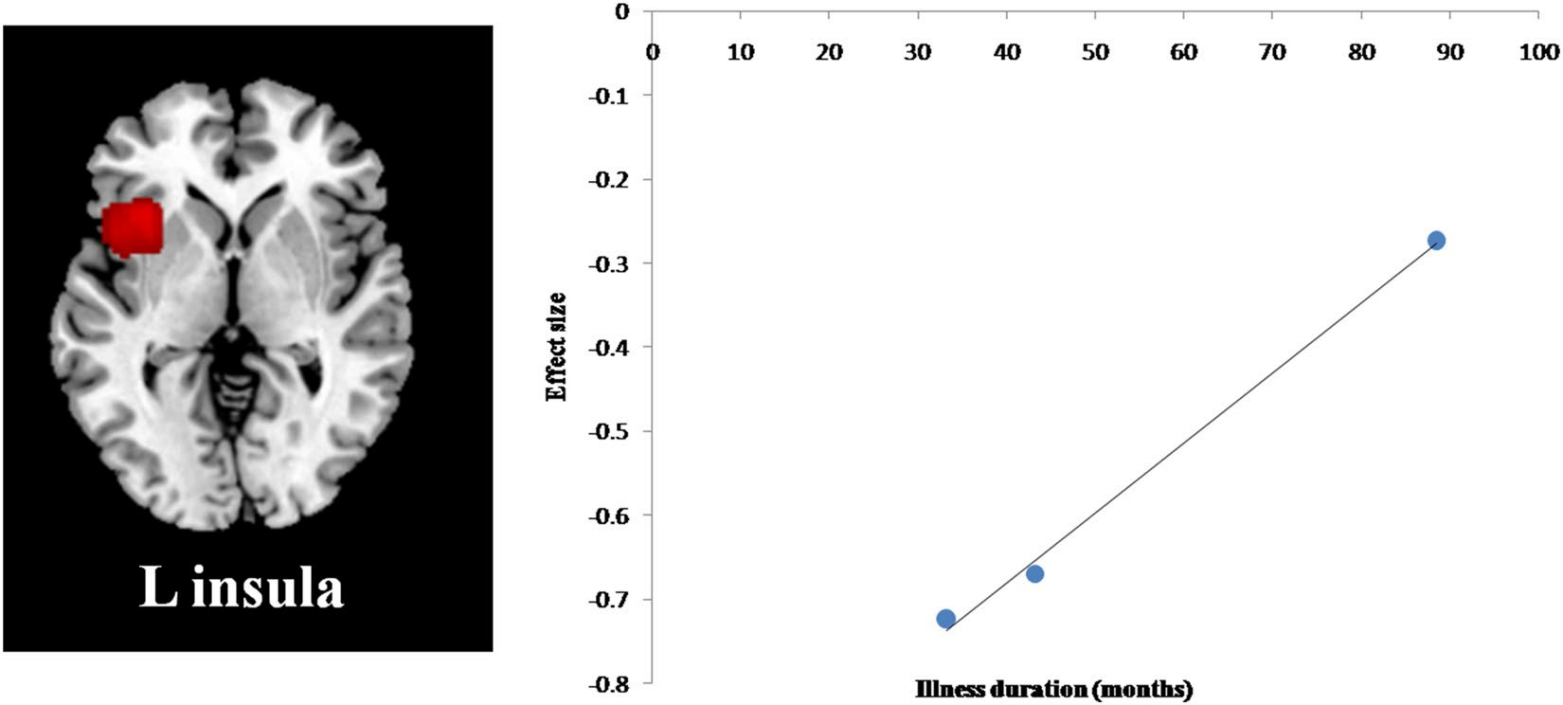
Association of grey matter volume in insula with illness duration in antidepressant responders compared with healthy controls, by meta-regression analysis.

We conducted meta-regression analyses to explore the association between GMV alteration and clinical data including the mean age, illness duration and the percentage of female patients in responders. Only GMV in the left insula showed positive correlation with illness duration (Fig. 2, peak MNI = −32, 16, 2 Z = 1.234, P = 0.0038, 113 voxels). However, this result should be interpreted with caution as it is driven by only three studies, and variability across studies might affect this analysis. Egger’s test of funnel plots in the responders (Figure S2) reveals asymmetry for the left insula (p = 0.105) and IFG (p = 0.534).

## Discussion

This meta-analysis is the first to analyze VBM studies in MDD antidepressant responders and nonresponders compared with healthy controls. GMV alterations in the cortico-limbic circuit, especially the prefrontal regions and ACC, were observed in both patient groups, implicating this in the intrinsic pathophysiology of major depressive disorder. However, there were notable differences between the patient groups. GMV in the bilateral ACC and right SFG was higher in responders, but lower in nonresponders. Lower GMV in the left insula and inferior frontal gyrus and higher GMV in right gyrus rectus were only observed in responders, and lower GMV in bilateral MCC was only observed in nonresponders, suggesting links to different mechanisms.

Generally, only responders showed higher GMV compared with healthy controls, while nonresponders showed broadly lower GMV. Previous neuroimaging studies have reported lower GMV in MDD^17, 24^, and postmortem studies have, accordingly, shown decreases in the density, number and size of neuronal and glial cells^25, 26^. Antidepressant drugs may restore GMV in responsive patients^27^, possibly through synaptic plasticity and altered expression of neurotrophic factors^28, 29^. This may explain why responders showed higher GMV while nonresponders showed lower GMV.

We found GMV differences in the bilateral ACC and right SFG, which were higher in responders but lower in nonresponders. This is consistent with reports that GMV is lower in SFG and that this correlates with the severity of depression^30, 31^ and that remission of depressive symptoms is associated with higher GMV in right ACC^32–34^, higher functional and metabolic activity in ACC^35–37^ and altered connectivity of the cingulate tracts^38^. As the major regions in the cortical-limbic network, bilateral ACC and right SFG are involved in dysfunctional mood and emotional regulation in MDD^39, 40^. Given that antidepressant medication influences brain structure mainly through the serotonergic system^41, 42^, GMV in bilateral ACC and right SFG might be especially sensitive to the clinical response to pharmacotherapy. Interestingly, a PET study found that nonresponders had lower serotonin transporter binding in ACC than responders^43^. A previous meta-analysis found the most robust grey matter reductions in a relatively focal region in rostral ACC, both in the pooled meta-analysis and in the subgroup analysis of multi-episode samples^44^, but not in subgroup analysis of first-episode studies^44^. A recent meta-analysis in first-episode depression also failed to find lower grey matter volume in the ACC^45^. As the symptoms of first-episode patients (and in our study, responders) are relatively lighter, this might suggest that ACC abnormalities are sensitive to the severity of symptoms in depressed patients.

In responders, GMV was lower in the left insula and IFG. Consistent with this are previous reports that lower GMV in bilateral insula is correlated with severity of depression^46^, and that GMV is lower in the left insula in current and remitted MDD^47^. Functional neuroimaging studies in MDD patients have implicated the insular cortex in the emotional processing of guilt and sadness^48, 49^. The lower GMV in the left insula in our study appears consistent with functional studies in MDD demonstrating lower activity in the bilateral or left-side insula^50, 51^. Although the role of the insula in the pathophysiological processing in MDD still need be clarified, our results suggest a positive involvement in the treatment response of MDD patients, as was also observed in medication-free MDD patients in a previous meta-analysis^52^. Furthermore, meta-analysis of studies of medication-free patients with MDD has also shown lower GMV in the IFG^52^. In a longitudinal functional neuroimaging study using near infrared spectroscopy (NIRS), both untreated and remitted MDD groups showed significantly lower [oxy-Hb] activation during a verbal fluency task in the bilateral prefrontal cortices compared to HC^53^. These findings may indicate that brain structure and function in the left insula and IFG remains impaired in remitted patients even after improvement of depressive symptoms.

The study has some limitations. First, the generalizability of the results was limited by the small sample size which combined only 5 nonresponder datasets and 9 responder datasets, which also meant that meta-regression could not be performed on the nonresponders. Second, important variables like intelligence quotient and handedness were not generally reported, precluding exploration of their impact on the results. Third, there is the potential for bias in the VBM method, whose relative insensitivity to spatially more diverse changes can lead to over-representation of group differences in regions of high anatomic variability. Fourth, the number of included studies was insufficient for an analysis of the effects of particular antidepressants or antidepressant classes. Fifth is the imprecision inherent in all methods depending on summarized coordinates, which are nevertheless necessary because published studies typically use different covariate models or raw statistics.

## Conclusion

Taken together, the present findings demonstrate structural grey matter differences in regions involved in cortico-limbic networks in MDD patients. GMV of the bilateral ACC and right SFG was lower in nonresponders, but higher in the responders, suggesting that this might provide biomarkers associated with antidepressant response and prolonged remission. Furthermore, lower GMV in the left insula and IFG was only present in responders. Longitudinal studies will need to investigate the dynamic effect of antidepressant medication, and for a better understanding of the underlying cause of these GMV alterations. In particular, this study adds to Psychoradiology (https://radiopaedia.org/articles/psychoradiology), an evolving su﻿bspecialty of radiology, which is primed to play a major clinical role in guiding diagnostic and therapeutic decisions in patients with mental disorders^54, 55^.

## Methods

### Inclusion criteria

We followed the Preferred Reporting Items for Systematic reviews and Meta-Analyses guidelines (PRISMA)^56^. We searched for recent studies published in Pubmed, Embase and Web of Science up to June 2016, using the keywords ‘depressive disorder’ or ‘unipolar depression’ or ‘depression’ or ‘depress*’ plus ‘VBM’ or ‘voxel-based morphometry’ or ‘voxel’ or ‘morphometry’. We also checked the reference lists of those articles for further researches.

Studies were included according to the following criteria: (1) a group of participants diagnosed as having MDD based on DSM criteria were compared with healthy controls; (2) VBM was used to analyze grey matter alteration in MDD patients; (3) coordinates were reported in a standard space like the Talairach space or the Montreal Neurological Institute (MNI) space; (4) the nonresponder group was defined as showing < 50% reduction in the 17-item Hamilton Depression Rating Scale (HDRS-17) total score (or Beck Depression Inventory or Montgomery and Åsberg Depression Rating Scale), and the responder group was defined as ≥50% reduction in the same scale, after treatment at a sufficient dose for 6 weeks.

Studies were excluded if they met the following criteria: (1) MDD patients were not compared with healthy controls; (2) coordinates were not clearly reported; (3) VBM was not used; (4) comorbid panic disorder was not excluded; (5) late-onset MDD patients or adolescents with MDD were enrolled.

### Study selection

Two investigators independently examined abstracts from the initial search, and disagreements were discussed with a third author to reach a consensus. Authors were blinded to the articles’ authors, their institutions and the source of funding in order to minimize potential bias. The full texts of studies thought to fulfill the inclusion criteria were assessed in detail to confirm eligibility.

### Data extraction

Two authors independently extracted data. Differences were resolved by discussion among the review authors. The following data were collected: first author’s name, year of publication, details of study design, patient characteristics (including gender, age, illness duration, and disease severity at baseline), sample size, agent dose, duration of treatment and changes in VBM. From each included study we chose the statistically significant peak coordinates of GMV differences resulting from whole brain analysis.

### Quality assessment

Based on previous studies^35, 57, 58^, the included studies were assessed for quality using a 13-point checklist, including clinical and demographic aspects as well as the imaging methodology. Two authors independently reviewed each paper and assigned a completeness rating for the following items: the quality of the diagnostic procedures, the demographic and clinical characterization, the sample size, the MRI acquisition parameters, the analysis technique and the quality of the reported results (see the Supplement, Table S1). Differences were resolved by discussion among the review authors and a consensus score was assigned, as presented in Table 1.

### Statistical Analysis

First, independent voxel-wise effects meta-analyses were conducted to investigate regional GMV differences within both responsive and non-responsive groups relative to controls. Second, subgroup analysis in responsive group was performed to investigate the GMV change after antidepressant treatment. Third, conjunction analysis was conducted to identify distinct brain regions where non-responders and responders differed from healthy controls; this used a multimodal analysis to compare the results from two independent meta-analysis^17, 59^. The AES-SDM method has been described in detail elsewhere^15^, and we only describe it briefly here. First, the peak coordinates of the brain regions that were significantly different at the whole-brain level were selected. To avoid a potential bias toward liberally thresholded regions, we checked all the included studies to ensure that the same threshold was used throughout the brain. Second, we separately recreated a standard Talairach map of the differences in grey matter for each study by using a Gaussian kernel. The recreation of the peak coordinates was based on converting the peak *t* value to Hedges’ effect size, and then applying a non-normalized Gaussian kernel to the voxels near the peak, which assigns higher values to the voxels closer to peaks. For null findings in the studies, the recreation was done with the same effect size, and all voxels in the effect size map were estimated to have a null effect size, which was the only difference. Similar to other effect sizes, the null effect size was also included in the random-effects meta-analytic models, thus modifying the meta-analytic effect size. Third, the mean of the study maps were analyzed using a voxel-wise calculation to generate a mean map, and this calculation was weighted by the square root of the sample size of each study, so a study with a larger sample size would contribute more. Finally, we used standard randomization tests to determine statistical significance, hence creating null distributions from which *p* values were directly obtained. The default AES-SDM kernel size and thresholds were used (full-width at half-maximum = 20 mm, voxel *p* = 0.005, peak height Z = 1, cluster extent = 10 voxels).

### Reliability analysis

The reliability of results was examined by using Jack-knife sensitivity analysis. The sensitivity analysis was repeated 5 times for nonresponder groups and 9 times for responder groups, to find highly replicable brain regions which were preserved throughout most combinations of the datasets.

### Meta-regression analysis and publication bias analysis

Meta-regression was used to explore which of the following moderator variables might be responsible for heterogeneity of the findings: mean age of patients, illness duration, and the percentage of female patients. In the absence of consistent depression-scale data, meta-regression to depression symptom severity was not feasible. The probability threshold was reduced to 0.0005 to decrease the finding of spurious associations as less as possible, and regions which are not in the main analysis were ignored. Effect size estimates of the significant clusters were extracted to examine publication bias by using Egger’s test and funnel plots.

## Electronic supplementary material

Supplementary Information
