# Supplementary material for: Brain grey matter volume alterations associated with antidepressant response in major depressive disorder
Source: Sci Rep. 2017 Sep 5;7:10464. doi: 10.1038/s41598-017-10676-5 (PMC5585337; doi:10.1038/s41598-017-10676-5)

**Brain grey matter volume alterations associated with antidepressant response in major depressive disorder: a meta-analysis**

Jia Liu^1, †^, Xin Xu^2, †^, Qiang Luo^1^, Ya Luo^2^, Ying Chen^2^, Su Lui^1^, Min Wu^1^, Hongyan Zhu^3*^, Graham J Kemp^4^, Qiyong Gong^1^

Table S1: Quality Assessment Checklist (When criteria were partially met, 0.5 points were assigned)

| **Category 1: Participants** | Score (0/0.5/1) |
| --- | --- |
| 1. Patients were evaluated prospectively, specific diagnostic criteria were applied, and demographic data were reported.  2. Healthy comparison participants were evaluated prospectively, psychiatric and medical illnesses were excluded.  3. Important variables (e.g., age, sex, illness duration, onset, medication status, intelligence quotient, i.e. IQ, handedness, severity of illness) were checked either by stratification or statistically.  4. Sample size per group > 10. | |
| **Category 2: Methods for image acquisition and analysis** | |
| 5. Whole brain analysis was automated with no a priori regional selection.  6. Magnet field strength > 1T.  7. MRI slice-thickness ≤ 3 mm and more than 1 slice was identified and traced.  8. Zero gap width.  9. Coordinates reported in a standard space.  10. The imaging technique used was clearly described so that it could be reproduced.  11. Measurements were clearly described so that they could be reproduced. | |
| **Category 3: Results and conclusions** | |
| 12. Statistical parameters for significant and important non-significant differences were provided.  13. Conclusions were consistent with the results obtained and the limitations were discussed. | |
| TOTAL /13 | |

**Fig. S1: Meta-analysis of voxel-based morphometry studies with and responsive and non-responsive MDD patients in comparison with HC.**

From: Moher, D., Liberati, A., Tetzlaff, J., Altman, D. G., & Grp, P. (2009). Preferred Reporting Items for Systematic Reviews and Meta-Analyses: The PRISMA Statement.Plos Medicine. 6(7), e1000097.

Abbreviation: HC = healthy controls.


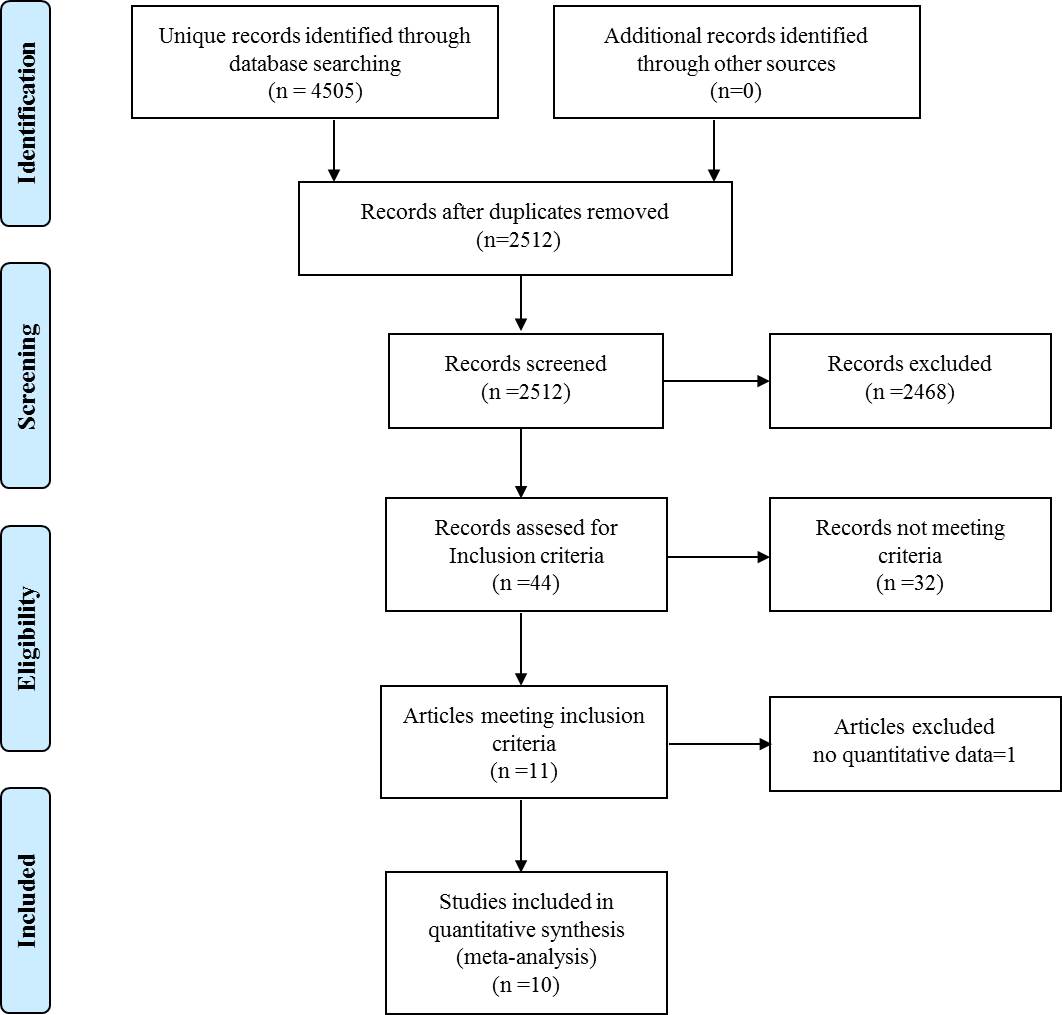


**Fig. S2: Funnel plots in responsive group**


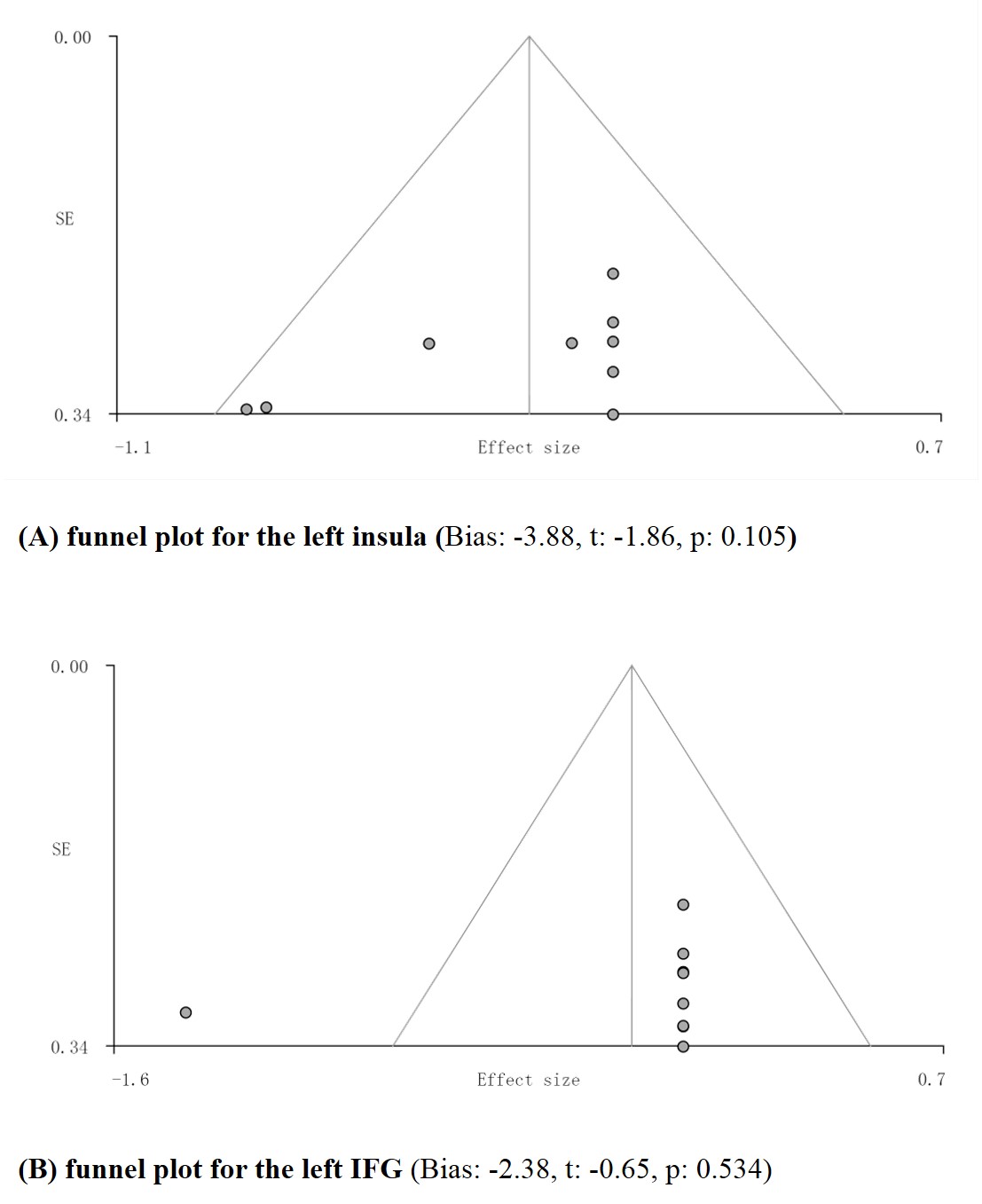

Supplement: Supplementary file 1 — Supplementary Information [file 41598_2017_10676_MOESM1_ESM.doc]
